# Evaluation of Optimal Vibrotactile Feedback for Force-Controlled Upper Limb Myoelectric Prostheses

**DOI:** 10.3390/s19235209

**Published:** 2019-11-28

**Authors:** Andrea Gonzalez-Rodriguez, Jose L. Ramon, Vicente Morell, Gabriel J. Garcia, Jorge Pomares, Carlos A. Jara, Andres Ubeda

**Affiliations:** Human Robotics Group, University of Alicante, 03690 Alicante, Spain; aerdna.gr@gmail.com (A.G.-R.); jl.ramon@ua.es (J.L.R.); vicente.morell@ua.es (V.M.); gjgg@ua.es (G.J.G.); jpomares@ua.es (J.P.); carlos.jara@ua.es (C.A.J.)

**Keywords:** vibrotactile actuation, sensory feedback, prosthetics

## Abstract

The main goal of this study is to evaluate how to optimally select the best vibrotactile pattern to be used in a closed loop control of upper limb myoelectric prostheses as a feedback of the exerted force. To that end, we assessed both the selection of actuation patterns and the effects of the selection of frequency and amplitude parameters to discriminate between different feedback levels. A single vibrotactile actuator has been used to deliver the vibrations to subjects participating in the experiments. The results show no difference between pattern shapes in terms of feedback perception. Similarly, changes in amplitude level do not reflect significant improvement compared to changes in frequency. However, decreasing the number of feedback levels increases the accuracy of feedback perception and subject-specific variations are high for particular participants, showing that a fine-tuning of the parameters is necessary in a real-time application to upper limb prosthetics. In future works, the effects of training, location, and number of actuators will be assessed. This optimized selection will be tested in a real-time proportional myocontrol of a prosthetic hand.

## 1. Introduction

An amputation is the removal of a limb caused by a trauma, a medical condition, or surgery. Negative impacts to the amputee include the loss of function and sensory perception of the limb as well as changes in their interaction with the environment that may lead to psychological conditions. Limb prosthetics are used to limit the effects of this trauma. A prosthesis can be defined as an artificial replacement of the lost limb to regain independence after the amputation. Active prostheses allow the user to interact with their environment, e.g., by opening and closing an artificial hand to grasp objects [1]. One of the most common control methods is the use of the residual electrical activity of the nerves measured on the stump as an input to the prosthesis. This kind of control is called myoelectric and it naturally replicates how healthy individuals control their limbs [2].

One of the main issues of upper limb myoelectric prostheses is the way of dealing with sensory feedback [3]. Open-loop prostheses only account for visual feedback of how the grasping is achieved, so users do not have precise information of grasping forces leading to difficulties in the manipulation of fragile objects. Indeed, most current commercial prostheses only provide feedforward control of grasping. To solve this problem, precise sensors can be used in the prosthetic hand to give back force information to the user during its operation [4,5]. The closed-loop approach can then be achieved in several ways. One way is to provide meaningful visual feedback of the exerted force, e.g., by adding visual force information on the prosthetic hand [6]. One way of addressing this approach is the use of virtual environments delivering information of a simulated upper limb [7]. The use of visual feedback of the actual neuromuscular processes that are taking place has emerged as an effective way to increase user involvement [8]. In a recent study, a novel visual feedback approach is proposed as a combination of force information with electromyographic biofeedback to enhance sensory perception [9]. Auditory feedback has been also applied alone or in combination with visual feedback [10].

Other methods are based on providing actual physical sensations to the user. Vibrotactile feedback is the most used method to provide force information during grasping tasks [11,12,13,14]. Vibrotactile actuators are lightweight and small and deliver tactile feedback through vibration patterns. Vibrotactile actuation can provide additional sensory information in a broad number of domains ranging from leisure activities to rehabilitation performance [15,16]. Designing an effective vibrotactile feedback system allows users to perceive and respond to force stimuli correctly. In the field of rehabilitation, vibrotactile feedback is commonly used for both upper and lower limb protheses [17,18]. Another similar approach is the use of mechanotactile actuation [19]. In contrast to vibrotactile feedback, this technology has a better resolution, making it easier to distinguish between different force levels, but it is heavier and larger. Force feedback can also be delivered through electrical stimulation [20,21]. However, this method can be painful and unpleasant to the user if the signal amplitude is too large. This is especially critical if stimulation is performed invasively [22].

Besides the effective introduction of force feedback in current commercial prostheses, one major aspect that still needs to be properly assessed is how well these previously described methods can discriminate between different force levels, i.e., given the method, how to provide the user with a robust, reliable and easy to embody force feedback approach. To date, several studies have dealt with the comparison of different types of feedback added to the control scheme [23]. This concept is generally defined as multisensory feedback [24]. However, little attention has been paid to a precise parameter tuning in the delivered patterns.

For the lower limb, a few studies have focused on determining how to select certain parameters of the vibrotactile feedback. For instance, aspects such as number of actuators and location, delivered frequencies or habituation to the stimulus have been assessed [25,26]. In general, studies on vibrotactile feedback use no more than five different feedback levels [27] and do not focus specifically on the type of stimulus pattern that is delivered. This is also common in other feedback approaches.

The main goal of this study is to evaluate the latter aspect in a well-established method such as vibrotactile actuation. To that end, we have assessed both the selection of actuation patterns and the effects of the selection of frequency and amplitude parameters to discriminate between different feedback levels that could be then assigned to different levels of the exerted force of the prosthesis. The experimental protocol is proposed as a tool for optimally selecting the best vibrotactile pattern to be used in a closed loop control of upper limb myoelectric prostheses.

## 2. Materials and Methods

**Figure 1:**
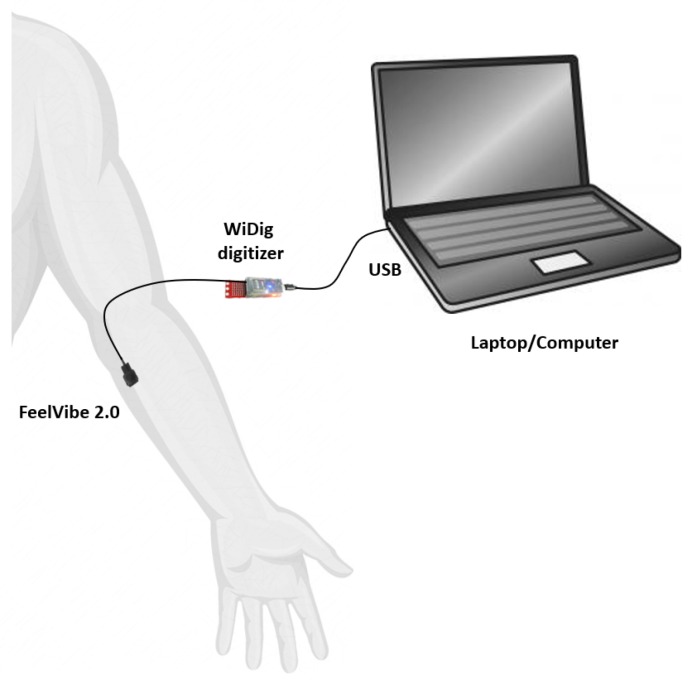
Experimental set-up.

Nine subjects participated in the study (6 male and 3 female aged 26.4±3.2 years old). All subjects were in perfect physical condition with no history of neurological disease. The experimental setup was very simple. Vibrations were delivered with different parameters to study the optimal vibrotactile pattern. The FeelVibe actuator (I-CubeX, Infusion Systems) is based on an Eccentric Rotating Mass (ERM) motor with a haptic driver. The actuator dimensions are 19 × 19 × 7 mm, making it ideal for placement in adequate positions of the amputated limb, either the arm or the stump itself. In the present study the actuator was placed on the forearm of the participants (see Figure 1) .

The FeelVibe actuator is connected to a digitizer WiDig with up to 8-channel capability, allowing for multiple vibrating sources. This digitizer is connected through USB to the computer and actuated using Touch Sense 2200 software with a VirtualMIDI driver, which allows delivering up to 123 predefined vibration patterns. From this set of patterns, different combinations have been selected to configure the experimental protocol.

**Figure 2:**
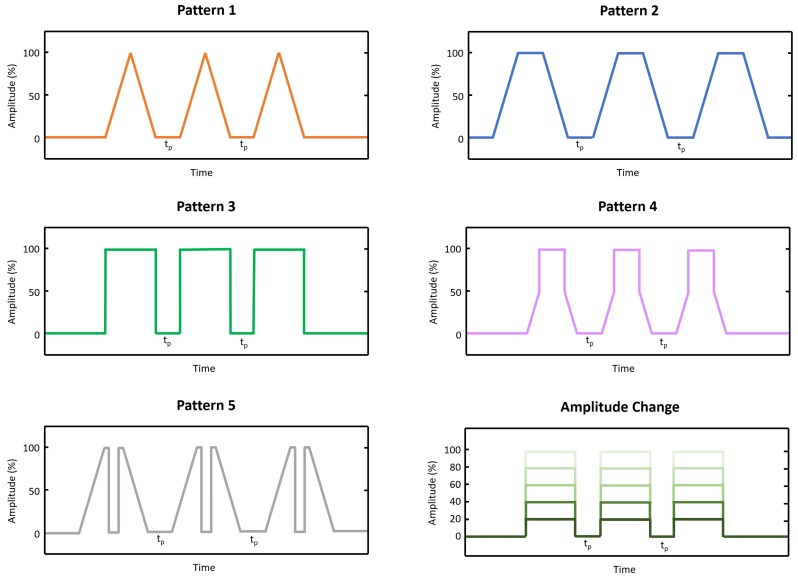
Vibration patterns delivered to the subjects.

Two different sets of vibrations were applied:

- **Pattern shape:** For this set, subjects were provided with vibrotactile feedback of different patterns. Feedback levels were simulated by increasing and decreasing the time lag (tp) between three consecutive vibration peaks. The vibrating frequency of the onset segments was fixed by the hardware and was felt by subjects as a continuous stimulus. The time lag was selected in a scale between 0.1 to 1 s in steps of 0.1 s, making a total of 10 different feedback levels. A maximum tp of 1 s was selected to avoid long response times in a future application of these patterns to a real-time prosthetic control. A total of 5 different patterns (see Figure 2) were evaluated. Each feedback level was repeated 5 times and randomly delivered to the subject making a total of 100 trials per pattern.
- **Pattern amplitude:** For this set, Pattern 3 was selected and amplitude was changed between 20% to 100% in steps of 20% making a total of 5 feedback levels. This pattern was selected as it was the only predefined pattern that could provide different amplitude levels. The number of feedback levels was limited to 5 due to the amplitude resolution provided by the device. Each feedback level was repeated 10 times and randomly delivered to the subject making a total of 50 trials.

Before starting the experiments, subjects were asked if they were capable of feeling differences between all consecutive feedback levels. We checked this with all subjects and all answered positive. Subjects were then asked to evaluate the patterns using two possible approaches: relative difference and absolute level. For the first one, subjects were asked to say if the current pattern was softer, stronger, or equal compared to the previous by voting +1, −1, or 0, respectively. The first vibration was not voted and served as reference for the remaining. In a second run, subjects were provided again with the same set of vibrations and were asked to determine the exact feedback level by voting from 1 to 10.

To record subject replies and easily deliver the selected patterns, a customized Matlab software has been implemented. This software communicates with WiDig digitizer through the serial port and allows saving all the experimental information in an *xls* file for future analysis.

## 3. Results

**Figure 3:**
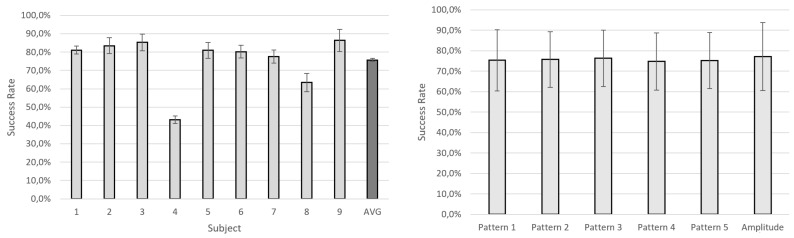
Average success rate (%) in the evaluation of the relative differences of pattern shapes (1 to 5) and pattern amplitude. Results per subject (**left**). Results per pattern (**right**).

**Figure 4:**
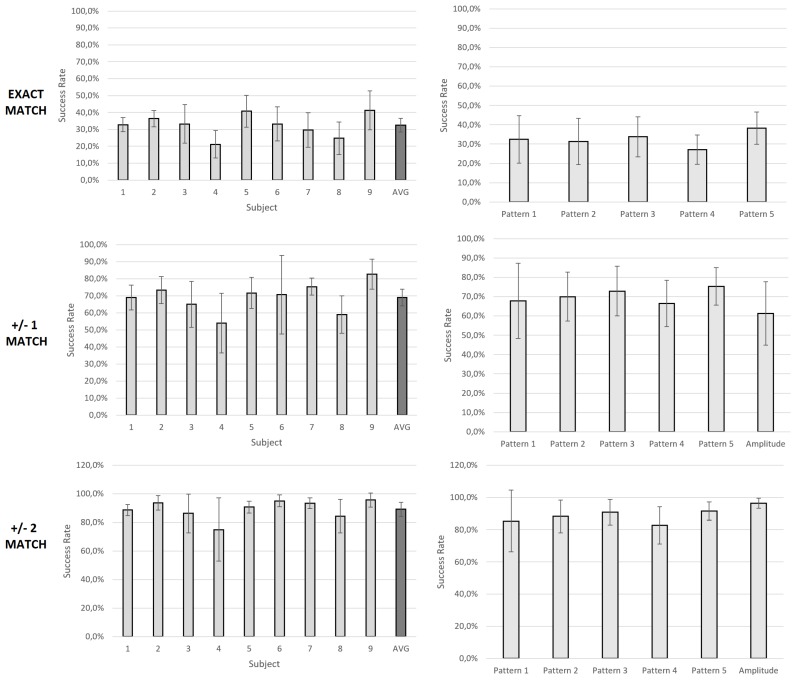
Average success rate (%) in the evaluation of the absolute feedback values for pattern shapes (1 to 5) and pattern amplitude. The first row represents exact matches in feedback level perception. The second row represents close matches (no further than 1 in error) in feedback perception. The third row represents further matches (no further than 2 in error) in feedback perception. Amplitude change success rate is only showed for the second and third row.

Figure 3 shows the success rate obtained for different patterns when taking into account relative difference between consecutive stimulations. Results are similar for most of the subjects, with an average of 75.7% ± 0.9% (Figure 3, left). Only subject 8 (63.4% ± 4.9%) and subject 4 (43.2% ± 2.1%) are significantly lower (Wilcoxon Signed-Rank Test, *p* < 0.05 with a Bonferroni–Holm correction). The worst performance of these subjects is stable for all the evaluated patterns. The average results per pattern indicate that all of them are similarly distinguished in their variation (Figure 3, right). Interestingly, changes in pattern frequency and changes in amplitude do not show significant differences in performance (Wilcoxon Signed-Rank Test, *p* > 0.05 with a Bonferroni–Holm correction). In Figure 4, success rates for an exact match of the feedback level are shown. The results show that subjects have a low accuracy in their perception of absolute values with an average of 32.6% ± 4.0% (Figure 4, top-left). This means that subjects can accurately perceive only around one third of the delivered feedback levels. As in the previous analysis, differences across subjects are not very high. Subjects 4 and 9 are again the lowest, with rates of 24.8% ± 9.7% and 21.2% ± 8.1%, respectively. However, these differences are, in this case, nonsignificant (Wilcoxon Signed-Rank Test, *p* > 0.05 with a Bonferroni–Holm correction). As for the previous approach, patterns 1 to 5 do not show significant differences (Wilcoxon Signed-Rank Test, *p* > 0.05 with a Bonferroni–Holm correction). Note that, in contrast to the relative difference approach, where intrasubject success rate was very stable, the standard deviation increases to ~10%, meaning that certain patterns are more difficult to be perceived by subjects. An illustrative example of this is Subject 4, who achieves very low accuracy for Pattern 1: an 8% compared to the remaining patterns where more than 20% accuracy is obtained.

To evaluate the convenience of using amplitude changes instead of frequency variation of the vibrotactile patterns, absolute matches have been computed again with a maximum error of one level only for patterns 1 to 5 (frequency change). This allows evaluating success rate of only 5 different levels to be compared to the 5 different amplitude levels delivered in the second set of vibrations. As expected, success rate increases to an average of ~69.0% ± 5.0% (Figure 4, bottom-left). This is very similar for all 5 patterns (Figure 4, bottom-right). However, success rate in feedback amplitude changes is slightly lower (61.4% ± 16.5%) but again they do not show significant differences (Wilcoxon Signed-Rank Test, *p* > 0.05 with a Bonferroni–Holm correction). An additional comparison has been made by increasing the possibility of error in perception to a maximum error of two. In that case, success rate increases to almost 100%. Differences between subjects and approaches continue to be nonsignificant but intrasubject accuracy across patterns is the same or even increases with very high deviations for particular subjects (4 and 8).

**Figure 5:**
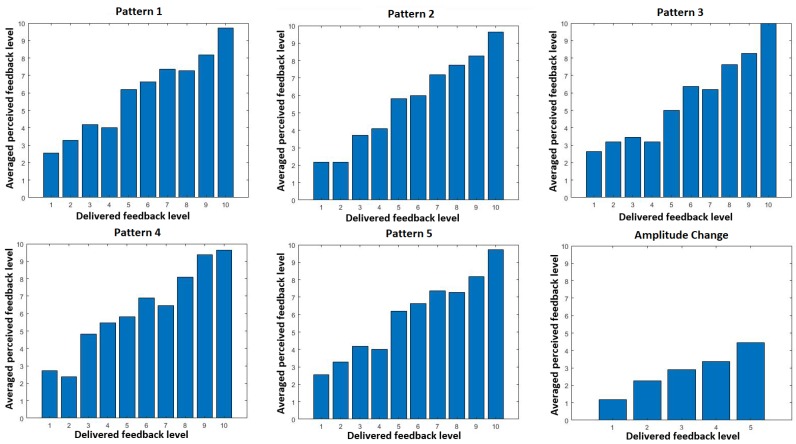
Correlation between perceived and delivered feedback levels for all 6 patterns.

For the last analysis, the correlation between perceived and delivered feedback has been evaluated by showing how well subjects selected the correct delivered feedback level in average (Figure 5). For all patterns, there is a high correlation 0.92% ± 0.04%. Patterns 1 to 5 show correlations above 0.9. However, amplitude changes are not so well correlated (0.82). From the graph, it can be clearly seen that high feedback levels are generally perceived as a lower feedback level. Another interesting aspect is that with a higher resolution in delivered feedback levels (patterns 1 to 5), subjects have more difficulties to perceive differences. In Figure 5, only the amplitude change curve (with only 5 levels) is increasing monotonically.

## 4. Discussion

One of the primary goals of this study was to select the best vibrotactile pattern to be used as feedback for the force control of an upper limb prostheses. To this end, different pattern shapes were evaluated discriminating feedback levels that corresponded to force levels. Stimulation trains were delivered to the subjects by varying the vibration frequency. The results show that the pattern shape has a small influence in the feedback level perception and there is no significant difference between the selected patterns (Figure 3 and Figure 4). Therefore, when tuning parameters of vibrotactile feedback, other factors need to be considered. In this study, we assume two different perspectives—frequency tuning or amplitude tuning—and, in this case, the frequency tuning approach led to a slightly better perception of feedback levels but nonsignificant. Previous studies show that high frequencies are better perceived than low frequencies [26], this may be the result of the fact that the amplitude and the frequency are coupled for ERM motors. One possible reason why these differences are not appreciated in our study is that changes in frequency of delivered trains reached a maximum of ~10 Hz and were much smaller that in [26], so the effect of the coupling was not present. Although further experiments must be done to evaluate this differences, the results suggest that in a real-tune application of vibrotactile feedback, delivered feedback levels are similarly perceived in terms of vibration frequency and amplitude level.

Another key factor is how many feedback levels are subjects capable of differentiating with enough accuracy. Figure 4 indicates that 10 different feedback levels are difficult to perceive (32.6% ± 4.0%). When this number of feedback levels is decreased to five, the success rate importantly increases (69.0% ± 5.0%), and it is even higher when up to two levels perception error is allowed (89.2% ± 4.9%). Determining the optimal number of levels to discriminate depends on the subject perception capabilities and the approach used to deliver difference in feedback levels (frequency or amplitude). A fine-tuning of these parameters is necessary to optimally apply this kind of sensory feedback. In fact, most of the current studies do not include more than five different feedback levels [27]. Proportional myoelectric control, in which a non-finite feedback level is needed, may benefit from a correct selection of the number of delivered feedback levels. Decrease in feedback perception was present for subjects 4 and 9. In those cases, subjects had more difficulties in discriminating feedback levels. This suggests that sensorimotor perception is very subject-dependent. Indeed, many patterns differ significantly for particular subjects that show a very high standard deviation. A possible way of reducing this perception error may come through a proper training protocol.

Our study is limited to a particular set of vibration patterns and tuning parameters. In future works, we will analyze effects of training or habituation, location, and number of actuators. Additionally, a comparison of different frequency and amplitude resolutions, i.e., number of perceived feedback levels, will be performed to define the maximum number of feedback levels that can be optimally distinguished. As previosly mentioned, these aspects have already been partially addressed in other studies for the lower-limb [25,26], but a systematic evaluation on the upper limb is necessary as a big number of prosthetic devices are used by transradial amputees. Location and number of actuators can be explored to evaluate effects in perception error, for instance, by adding additional vibration trains in different body parts or changing their location to a more sensitive area. An interesting approach to this issue has been evaluated in [25], where an array of multiple actuators is places from the proximal to the distal part of the thigh. Others factors not included in previous studies are the effects of a continuous feedback versus a discrete one and the suitability of the proposed patterns in terms of subject time response, which is a critical factor in closed loop control. To evaluate the tuning of vibrotactile parameters, real-time proportional myocontrol of a prosthetic hand will be combined with vibrotactile sensory feedback. Vibrotactile patterns will be delivered proportional to the exerted force of the robotic hand measured from force sensors. This will help to determine how well a properly tuned feedback increases grasping accuracy and force perception. In this context, factors such as the influence of the socket in the perception of the vibration patterns can as well be examined.
